# Familial Cerebellar Ataxia and Amyotrophic Lateral Sclerosis/Frontotemporal Dementia with *DAB1* and *C9ORF72* Repeat Expansions: An 18‐Year Study

**DOI:** 10.1002/mds.29221

**Published:** 2022-09-23

**Authors:** Angela Rosenbohm, Hendrik Pott, Mirja Thomsen, Haloom Rafehi, Sabine Kaya, Silke Szymczak, Alexander E. Volk, Kathrin Mueller, Isabel Silveira, Jochen H. Weishaupt, Holger Tönnies, Philip Seibler, Katja Zschiedrich, Susen Schaake, Ana Westenberger, Christine Zühlke, Christel Depienne, Joanne Trinh, Albert C. Ludolph, Christine Klein, Melanie Bahlo, Katja Lohmann

**Affiliations:** ^1^ Department of Neurology University of Ulm Ulm Germany; ^2^ Institute of Neurogenetics University of Lübeck Lübeck Germany; ^3^ Division of Population Health and Immunity The Walter and Eliza Hall Institute of Medical Research Parkville Australia; ^4^ Department of Medical Biology The University of Melbourne Parkville Australia; ^5^ Institute of Human Genetics University Hospital Essen Essen Germany; ^6^ Insitute of Medical Biometry and Statistics University of Lübeck Lübeck Germany; ^7^ Institute of Human Genetics University Medical Center Hamburg‐Eppendorf Hamburg Germany; ^8^ i3S‐Instituto de Investigação e Inovação em Saúde Universidade do Porto Porto Portugal; ^9^ Division of Neurodegeneration, Neurology Department University Medicine Mannheim, Heidelberg University Mannheim Germany; ^10^ Institute of Human Genetics Christian‐Albrechts‐University Kiel Germany; ^11^ Institute for Human Genetics University of Lübeck Lübeck Germany; ^12^ German Center for Neurodegenerative Diseases, Site Ulm Ulm Germany

**Keywords:** spinocerebellar ataxia, linkage studies in genetics, ALS, frontotemporal dementia, SCA37, DAB1, repeat expansion, genome sequencing, nanopore sequencing

## Abstract

**Background:**

Coding and noncoding repeat expansions are an important cause of neurodegenerative diseases.

**Objective:**

This study determined the clinical and genetic features of a large German family that has been followed for almost 2 decades with an autosomal dominantly inherited spinocerebellar ataxia (SCA) and independent co‐occurrence of amyotrophic lateral sclerosis (ALS) and frontotemporal dementia (FTD).

**Methods:**

We carried out clinical examinations and telephone interviews, reviewed medical records, and performed magnetic resonance imaging and positron emission tomography scans of all available family members. Comprehensive genetic investigations included linkage analysis, short‐read genome sequencing, long‐read sequencing, repeat‐primed polymerase chain reaction, and Southern blotting.

**Results:**

The family comprises 118 members across seven generations, 30 of whom were definitely and five possibly affected. In this family, two different pathogenic mutations were found, a heterozygous repeat expansion in *C9ORF72* in four patients with ALS/FTD and a heterozygous repeat expansion in *DAB1* in at least nine patients with SCA, leading to a diagnosis of *DAB1*‐related ataxia (ATX‐DAB1; SCA37). One patient was affected by ALS and SCA and carried both repeat expansions. The repeat in *DAB1* had the same configuration but was larger than those previously described ([ATTTT]_≈75_[ATTTC]_≈40‐100_[ATTTT]_≈415_). Clinical features in patients with SCA included spinocerebellar symptoms, sometimes accompanied by additional ophthalmoplegia, vertical nystagmus, tremor, sensory deficits, and dystonia. After several decades, some of these patients suffered from cognitive decline and one from additional nonprogressive lower motor neuron affection.

**Conclusion:**

We demonstrate genetic and clinical findings during an 18‐year period in a unique family carrying two different pathogenic repeat expansions, providing novel insights into their genotypic and phenotypic spectrums. © 2022 The Authors. *Movement Disorders* published by Wiley Periodicals LLC on behalf of International Parkinson and Movement Disorder Society.

Neurodegenerative diseases include several forms of movement disorders and dementia and are characterized by a broad clinical spectrum with sometimes overlapping features, which can make it difficult or even impossible to make a correct diagnosis. The autosomal dominant spinocerebellar ataxias (SCAs) comprise a highly heterogeneous group of rare movement disorders characterized by progressive cerebellar ataxia variably associated with pigmentary retinopathy, ophthalmoplegia, dementia, seizures, pyramidal and extrapyramidal signs, lower motor neuron signs, or peripheral neuropathy.^1^, ^2^ Pathogenic variants in about 30 genes have been linked to autosomal dominant forms of cerebellar ataxias.^3^ The mutational spectrum is broad and includes different types of repeat expansions.

Since discovering pathogenic repeat expansions in neurodegenerative diseases about 30 years ago, the list of genes with pathogenic repeat expansions has grown to more than 50 (reviewed in Depienne and Mandel^4^). The disease‐causing mechanism of repeat expansions is manifold and often not well understood. It can include gain‐ or loss‐of‐function mechanisms at the protein level, toxic RNA gain‐of‐function, non‐ATG‐initiated translation peptides, or transcriptional dysregulation.^4^, ^5^ Repeat expansions have recently regained increased interest due to the development of novel sequencing technologies and improved analysis tools,^6^ leading to the discovery of several novel ataxia‐linked repeat expansions such as in *RFC1* ^7^, ^8^ and *DAB1*.^9^

A heterozygous, repetitive ATTTC motif insertion in a polymorphic ATTTT repeat in an intron of the 5′‐untranslated region of *DAB1* has been shown to cause autosomal dominant SCA type 37 (SCA37; ATX‐DAB1).^1^, ^9^ Although the nonpathogenic alleles comprise seven to 400 ATTTT sequence repeats, pathogenic alleles have a rather complex and variable structure with the configuration ([ATTTT]_60‐79_[ATTTC]_31‐75_[ATTTT]_58–90_).^9^, ^10^ Based on two reports on a few families from Portugal and Spain, ATX‐DAB1 is characterized by a pure cerebellar syndrome distinctly presenting with dysarthria and, in a subset of patients, by altered vertical eye movements.^1^, ^9^ Repeat expansions in *DAB1* seem to be a relatively rare cause of SCA.^11^

We here report an 18‐year clinical and genetic follow‐up of a large German family with amyotrophic lateral sclerosis (ALS)/frontotemporal dementia (FTD) and SCA. A core family of two patients with ALS and FTD has previously been reported with affected members carrying a heterozygous, presumably pathogenic variant in the dynactin 1 (*DCTN1*) gene.^12^ We have now extended this family to a seven‐generation pedigree with 30 definitely affected members with either ALS/FTD or SCA with occasional additional extrapyramidal features and cognitive decline. Although the ALS/FTD phenotype is caused by a GGGGCC hexanucleotide repeat expansion in *C9ORF72*, an ATTTC repeat insertion in a large ATTTT repeat in *DAB1* was found to be the cause of the SCA phenotype in the other patients, allowing for a diagnosis of ATX‐DAB1 (*SCA37* locus).

## 1 Methods

### 1.1 Pedigree Study

**FIG 1:**
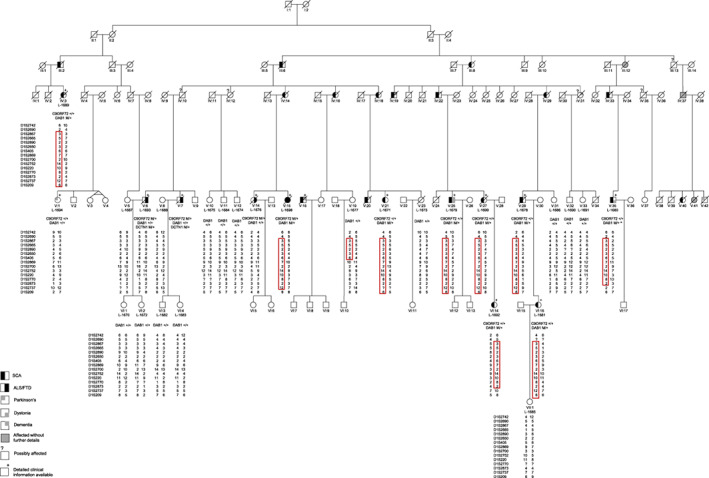
Pedigree of the family. Squares and circles represent males and females, respectively. Dashed symbols indicate individuals who are deceased. Clinical information is indicated as described in the legend. The “+” in the upper right corner of the symbol indicates individuals with detailed clinical information about neurological deficits. The “?” in the upper left corner indicates a possibly affected individual. “L” numbers represent individuals for whom DNA was available. Genetic status is given below the respective symbol, whereas “M” stands for the mutated allele and “+” for the wild‐type allele. Furthermore, the haplotype at 15 short tandem repeat markers in the *DAB1* region is indicated, and the shared haplotype among affected family members in highlighted by a red box. ^a^There was no DNA left for the repeat‐spanning long‐range polymerase chain reaction, thus the mutational status is based on the shared haplotype in the linked *DAB1* region. SCA, spinocerebellar ataxia. [Color figure can be viewed at wileyonlinelibrary.com]

The family pedigree was constructed based on genealogical information obtained by personal interviews of 10 family members and comprised 118 individuals (Fig. 1). Detailed medical information was obtained on 14 subjects by personal examinations (n = 11), telephone interviews (n = 3), and/or by medical records (n = 12) as indicated in Table 1. For five family members, the clinical status was set as possible SCA because it could not be established with certainty. No reliable information could be collected on the six ancestors in generations I and II.

**TABLE 1:** Clinical and genetic information on 14 investigated affected family members

	Pedigree number													
	IV:3 L‐1689	V:1	V:6 L‐1693	V:7	V:14 L‐1676	V:15 L‐1698	V:16, no DNA sample	V:21 L‐1671	V:25 L‐1679	V:27 L‐1699	VI:14 L‐1692	V:29 L‐1678	V:35 L‐1680	VI:16 L‐1681
Demographic information														
Age at examination (e) or age at death (d), years	≈80^d^	≈60^e^	≈70^d^	≈60^d^	≈80^d^	≈70^d^	≈70^d^	≈60^e^	≈70^e^	≈70^d^	≈40^e^	≈80^d^	≈70^e^	≈60^e^
Sex	Female	Female	Male	Male	Female	Female	Male	Female	Male	Female	Female	Male	Male	Female
Genetic information														
*DCNT1* variant			Yes	Yes										
*C9ORF72* expansion	No	No	Yes 6800–10,000 repeats	Yes 1300–1800 repeats	Yes >1300 repeats	Yes 7800–11,200 repeats	N.A.	No	No	No	No	No	No	No
*DAB1* expansion	Yes	No	No	No	No	Yes	N.A.	Yes	Yes	Yes	Yes	Yes	Yes	Yes
Acquisition of information														
Personal examination	X		X	X	X	X			X	X	X	X	X	X
Telephone interview		X				X		X						
Medical records		X	X	X	X	X	X	X	X	X	X		X	X
Clinical information														
Age of onset, years	15	60	61	54	77	66 (SCA) 67 (ALS)	35	49	40	32	22	40	27	55
Disease duration at examination or death, years	68	2	5	6	1.3	3 (SCA) 2 (ALS)	32	13	28	37	13	42	41	8
Predominant phenotype	SCA	atypical PD	FTD	ALS	ALS	ALS + SCA	SCA	SCA	SCA	SCA	SCA	SCA	SCA + DYT	SCA
Cortical dementia	X						X		X					
Frontotemporal dementia			X											
Gait ataxia	X					(X)	X	X	X	X	X	X	X	X
Arm ataxia	X					X	X	(X)	X	X	(X)	X	X	X
Altered horizontal eye movements (nystagmus, dysmetric saccades)	X					X	X	X	X	X	X	X	X	X
Vertical nystagmus							X		X	X	X		X	
Diplopia							X		X		X	X	X	
Dysarthria	X					X	X	X	X	X	X	X	X	
Dystonia									X				X	
Parkinson syndrome		X												
Arm tremor	X													
Upper motor neuron impairment				X	X									
Lower motor neuron impairment				X	X	X						X		
Reduced vibration sense						X						X		
SARA/INAS in patients with SCA	37/2					5/1	40/5	9.5/1	24/4	20/2	3/0	34/5	26/4	7/2
Brain MRI														
Not performed	X											X		
Normal		X			X									
Cerebellar atrophy						X	X	X	X	X	X		X	x
Frontotemporal atrophy			X											
Frontoparietal atrophy							X							
T2‐hyperintensity of pyramidal tract				X										
Atrophy generalized					X									
Hydrocephalus							X							
Brain PET														
Fluoro‐DOPA: symmetrical reduction in putamina			X											
F‐deoxy‐glucose: reduction in frontal and temporal lobes		X												

Abbreviations: N.A., not available; SCA, spinocerebellar ataxia; ALS, amyotrophic lateral sclerosis; DCNT1, Dynactin subunit 1PD, Parkinson's disease; FTD, frontotemporal dementia; SARA, Scale for the Assessment and Rating of Ataxia (score 0–40); INAS, Inventory of Non‐Ataxia Signs (count 0–16); MRI, magnetic resonance imaging; PET, positron emission tomography; DOPA, dihydroxyphenylalanine; a, age at death.

Diagnoses were established clinically with a particular focus on cerebellar signs. A diagnosis of ALS was made after excluding other causes of motor neuron disorders/mimics and according to the El Escorial criteria.^13^ An FTD diagnosis was made upon neuropsychological evaluation, brain imaging, and after the exclusion of other forms of dementia. The detailed neuropsychological examination included the following questionnaires and scales: Structured Interview for the Diagnosis of Dementia, Digit Span and Reverse Digit Span, Corsi−/ Block‐Tapping‐Test, Word Pairs from the Wechsler Memory Scale, German Adaptation of California Verbal Learning Test, Stroop Test, Verbal Fluency Benton, Picture Arrangement from the Wechsler Adult Intelligence Scale, and Tower of Hanoi. Six members affected by SCA (IV:3, V:25, V:29, V:35, VI:14, VI:16) underwent videotaping (examples are shown as Supplementary Material). Secondary causes of cerebellar ataxia and motor neuron syndrome mimics were excluded by neurological examination, laboratory testing, structural brain magnetic resonance imaging (MRI), and positron emission tomography (PET) (Table 1). MRI acquisitions for atrophy and structural alterations were performed on various 1.5 Tesla MRI scanners for diagnostic reasons. Two family members underwent positron emissions tomography: V:1 underwent F‐DOPA (dihydroxyphenylalanine) PET (200 MBq [18 F] DOPA was injected after 6 hours of fasting, and PET scans were performed to compare F‐DOPA accumulation in the putamen and nucleus caudatus compared with the occipital cortex). Neuroimaging for frontotemporal dementia was performed in V:6 by 18 F‐fluorodesoxyglucose‐PET to evaluate regional metabolism in brain areas, especially the frontal and temporal lobes. The ratio of the mean FDG‐PET concentration in a given area were compared with brainstem uptake.

### 1.2 Genetic Testing

The study was approved by the ethical board of the University of Ulm, Germany (file reference no. 20/10). Written informed consent was obtained from all participants (or guardians of participants). DNA was extracted from ethylene diamine tetraacetic acid–containing venous blood samples from 30 family members, including three ALS/FTD, one ALS + SCA, and eight SCA affected according to standard procedures. One patient passed away before genetic testing, but detailed medical records were available.

Genetic investigations in this family were manifold and subsequently applied over time.

#### 1.2.1 Testing for the cause of ALS/FTD

First, because two affected family members carried a presumably pathogenic variant in *DCTN1*,^12^ we tested for segregation of this variant in the other family members. Second, we tested for linkage to the *GRN* gene linked to FTD in 2006.^14^ Third, we performed a sequence analysis of all coding exons of the ALS‐linked genes *TARDBP (TDP43)* ^15^ in 2008 and *FUS* ^16^, ^17^ in 2009. Fourth, we tested for *C9ORF72* repeat expansions in clinically affected members with ALS/FTD and SCA. *C9ORF72* was added to the list of ALS/FTD genes in 2011.^18^, ^19^ The genetic analysis was performed as described, including Southern blot analysis to determine the number of repeats in carriers of expansions in *C9ORF72*.^18^, ^20^

#### 1.2.2 Testing for the cause of SCA

Initially, we tested two patients with SCA (IV:3, V:21) for known genetic causes of SCA. This included trinucleotide repeat expansions at the *SCA1*, *SCA2*, *SCA3*, *SCA6*, *SCA7*, *SCA8*, *SCA10*, *SCA12*, *SCA17*, *DRPLA*, and *FRDA* loci and sequencing of exon 4 in *PRKCG* (SCA14). Linkage analysis was also performed to test for a role of the *SCA4*, *SCA5*, *SCA13*, and *SCA27* loci. After excluding known causes, we performed a genome‐wide linkage analysis in 20 family members using 375 microsatellite markers. Two‐point and multipoint parametric logarithm of the odds (LOD) scores were calculated assuming autosomal dominant mode of inheritance and 95% penetrance using the software Allegro 2.0(deCODE genetics, Reykjavik, Iceland) by splitting the family in two branches (due to size limitations, data not shown). Regions with suggestive linkage underwent fine mapping using additional microsatellite markers. In a region with a shared haplotype (Fig. 1), linkage analysis was performed using 15 microsatellite markers for the whole family branch founded by II:3. Genetic distances were derived from the Rutgers (Rutgers University, Piscataway, NJ) Combined Linkage‐Physical Map version 3 (Kosambi, in centi‐Morgan, cM). Marker D1S2770 was not available in the Rutgers map, and genetic distance was interpolated using the Map Interpolator (http://compgen.rutgers.edu/map_interpolator.shtml) based on genomic position extracted from Ensembl (hg19) (European Molecular Biology Laboratory's European Bioinformatics Institute at the Wellcome Genome Campus in Hinxton, United Kingdom). The affection status was set to “affected” for SCA‐affected family members, to “unaffected” for healthy married‐ins, and to “unknown” for all other family members to take possible reduced penetrance into account. Model‐based linkage analysis was performed assuming autosomal dominant inheritance with one liability class with reduced penetrance (0.95) and no phenocopies. The frequency of the disease allele was set to 0.0001. Equal allele frequencies were assumed for each marker. Two‐point linkage and multipoint linkage analyses were performed using FASTLINK^21^ (version 4.1P) and SimWalk2^22^ (version 2.91), respectively. Software‐specific input files were generated using Mega2^23^ (6.0.0).

To rule out the presence of large chromosomal deletions/multiplications as a potential cause, we used array comparative genomic hybridization analysis with the Agilent (Santa Clara, CA) 244 k chip to conduct a genome‐wide search. Genome sequencing was carried out at the service provider KNOME (Cambridge, MA) in 2011 in V:25 and VI:14 with a mean coverage of 36x and 39x, respectively, which eventually identified the genetic cause after a reanalysis of the data in 2020. For genome data interpretation, we focused on the linked region on chromosome 1, looked for coding variants and repeat expansions using exSTRa^24^ and ExpansionHunter^25^ as described,^26^ and tested for overlap with newly reported SCA loci.

#### 1.2.3 Validation of a repeat expansion in *DAB1*

After the identification of a possible expansion of the ATTTT motif in *DAB1* and an unexpected high load of ATTTC‐repeat‐containing reads in the genome data of VI:14, we used repeat‐spanning, long‐range (LR) polymerase chain reaction (PCR) and repeat‐primed (RP) PCR as described^27^ but with the PrimeSTAR GXL DNA polymerase (Takara, Shiga, Japan). Furthermore, we applied Nanopore sequencing to confirm the repeat expansion and determine the repeat size and structure using expansion‐spanning LR‐PCR products as template generated using the KOD polymerase (EMD Millipore, Darmstadt, Germany). In brief, for the PCR amplicon Nanopore sequencing, 1 μg of DNA per product was used. Because of the known variability of Nanopore sequencing, we carried out a total of three runs. In the first run, family members V:21 and VI:14 were analyzed using the native barcoding kit EXP‐NBD104. In a second and third run, all available samples (n = 8) were multiplexed together using the newest Q20+ chemistry and native barcoding kit EXP‐NBD112‐24 or EXP‐NBD104. After library generation, samples were run on an R9.4.1 flow cell with >800 available pores at the start of sequencing on a GridION machine (Oxford Nanopore Technologies, Oxford, UK). High‐accuracy mode base‐calling was performed with the integrated *Guppy* algorithm (6.0.7) in MinKNOW (version 22.03.4)Oxford Nanopore, Oxford, United Kingdom. Both FAST5 and FASTQ files were generated through MinKNOW in real time and used for downstream bioinformatics analysis. All reads were mapped to the reference sequence with the software *Minimap2* (version 2.17)Harvard University, Boston, MA, USA. Samtools (version 1.9)England. was used for coverage determination and filtering. Motif mismatch detection was achieved with Noise‐Cancelling Repeat Finder (NCRF) (version 1.01.02)^28^ on reads with >900 bp to filter out the wild‐type allele.

## 2 Results

### 2.1 Clinical Findings

A total of 30 family members were definitely affected with a neurodegenerative disease, and another five were possibly affected (Fig. 1). Phenotypes comprised ALS (n = 3), FTD (n = 1), or SCA (n = 20). Two family members had a parkinsonian syndrome (Tables 1 and 2). One person (V:15) initially suffered from SCA and later developed ALS.

**TABLE 2:** Clinical information about deceased (possibly) affected family members obtained by family history interview

Pedigree number	Age of onset, years	Age at death, years	Clinical information	Estimated neurodegenerative disease as from family history
**III:2**	≈40	ND	“Multiple sclerosis” or “syphilis,” disturbance of balance	SCA
**III:6**	ND	≈70	Disturbance of balance, speech normal, mild form of ataxia, died during war with a heart attack	SCA
**III:8**	ND	ND	Unable to walk, had to be pushed in a wheelbarrow	SCA
**III:12**	ND	ND	Affected, but no detailed information	ND
*III:13*	ND	ND	Possibly affected, but no reliable information available	ND
*IV:10*	ND	≈40	Nervous person; died in accident	ND
*IV:12*	ND	≈80	“Mysterious sickness” in the last years	ND
**IV:14**	≈20	≈50	Slowly progressive difficulty walking, skiing, and ladder climbing with repeated falls, disturbance of balance; died from colon cancer	SCA
**IV:16**	≈35	≈70	From ≈35 years of age, she developed cerebellar ataxia, oculomotor palsy, gaze‐evoked nystagmus, saccadic eye movements, and a cerebellar dysarthria	SCA
**IV:18**	≈55	≈70	Difficulty walking, walked like being drunk, and muscle weakness, known as “Nonne Pierre Marie“ disease	SCA
**IV:19**	ND	ND	Dementia, small‐stepped gait	SCA
**IV:22**	ND	≈70	Ataxia (similar to his affected son)	SCA
**IV:29**	ND	≈~90	At age 20 years, was bedridden with ataxia with previous difficulty walking, no muscle weakness, other signs similar to her affected son	SCA
*IV:31*	≈80	≈90	Severe dementia (known as Alzheimer's disease) with urine incontinence, mutism, swallowing problems, bedridden	Alzheimer's disease
**IV:33**	≈50	≈80	Small‐stepped gait, ataxia, speech disturbance, similar to V:35	SCA
*IV:35*	ND	ND	Difficulty walking in the last years	ND
**IV:37**	ND	ND	Affected, but no detailed information	ND
**V:20**	ND	≈30	Difficulty walking, heart disease	SCA
**V:40**	ND	ND	Cerebellar ataxia similar to V:16	SCA
**V:41**	ND	ND	Affected, but no detailed information	ND
**V:42**	ND	ND	Parkinsonism	Parkinson's disease

Bold pedigree number indicates definitely affected family member; italic pedigree number indicates possibly affected family member.

Abbreviations: ND, not determined; SCA, spinocerebellar ataxia.

Detailed clinical information was obtained for 14 affected individuals (Table 1, Supplementary Material). Of these patients, three presented with a pure SCA (V:27, VI:14, VI:16) and eight with predominant SCA and additional neurological deficits including dementia (n = 3), oculomotor palsy (n = 6), dystonia (n = 2), tremor (n = 1), and/or sensory deficits (n = 2). One examined person had isolated atypical parkinsonism (V:1). Four examined family members were affected with FTD (n = 1) or ALS (n = 3); one patient had both ALS and SCA (V:15). One of the patients with SCA (V:29) showed sensory involvement with reduced vibration sense, distal numbness, and flaccid proximal accentuated (nonprogressive) motor tetraparesis after decades of SCA but no classical ALS. He was the only patient with SCA with dysphagia, which developed 40 years after SCA onset. None of the patients reported oscillopsia or showed saccadic intrusions.

The cerebellar syndrome in our patients comprised a pronounced gait ataxia (n = 10/10), mild ataxia affecting the upper limbs (n = 10/10), and cerebellar dysarthria (n = 9/10). Regarding the early‐altered vertical eye movements that had been reported in the Spanish ATX‐DAB1 families,^10^ in our family, abnormal horizontal eye movements were a common sign, but vertical gaze nystagmus was noted in five of 10 patients with SCA only. Because vertical eye movement disturbances are assumed to be an early sign,^10^ it should be noted that the five affected individuals without vertical eye movement alterations had a disease duration of 2 to 66 years at examination.

Information on another 16 definitely affected and five possibly affected family members was obtained by family history interview and revealed a variety of motor and cognitive problems (Table 2).

Age at onset (AAO) in patients with SCA was at 38.7 ± 14.4 years (range, 15–66 years; n = 15), and the condition progressed slowly for several decades. For the ALS/FTD phenotype, AAO was 64.8 ± 9.7 years (range, 54–77 years; n = 4), with a mean disease duration of 2.5 years. The patient with ALS and SCA (V:15) had a decline of motor function for 2 years. Men and women were affected at comparable frequencies and showed no apparent differences in clinical symptoms such as AAO in patients with SCA (women, 38.8 ± 18.1 years; men, 38.7 ± 7.5 years).

Notably, there was no evidence of obvious anticipation of the SCA phenotype because available parent–child pairs had a variability of AAO in both directions: a lower AAO was observed in five offspring (6–25 years), but a later AAO was found in three offspring (15–57 years) and equal AAO in one offspring when compared with their affected parent.

**FIG 2:**
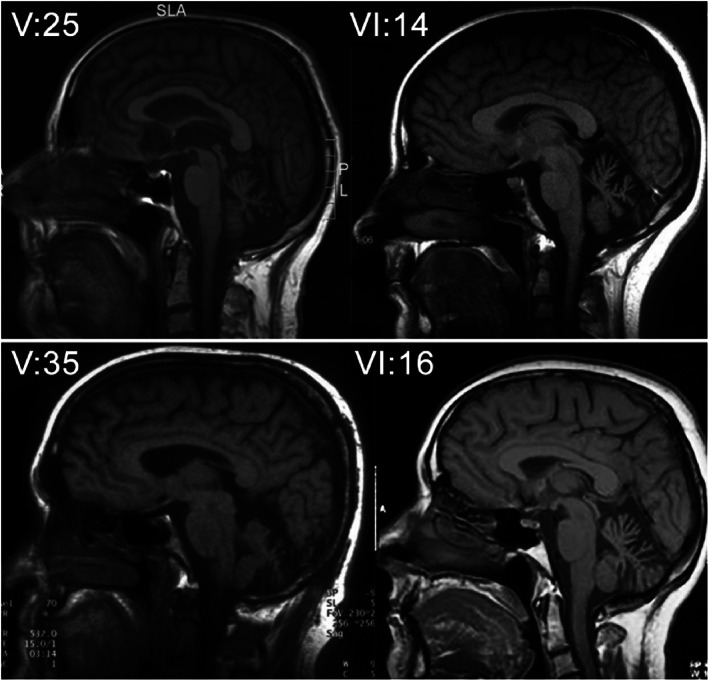
Brain imaging. Midline sagittal T1‐weighted brain magnetic resonance imaging of four family members (V:25, VI:14, V:35, VI:16) with spinocerebellar ataxia and cerebellar atrophy. V:25 presents with atrophy of the cerebellar vermis and the cerebellar hemispheres (age, 69 years; duration of disease, 29 years). VI:14 also shows cerebellar atrophy, particularly evident in the superior vermis (age, 30; duration of disease, 8 years). In V:35, atrophy of the cerebellar hemispheres was evident (age, 60 years; duration of disease, 33 years). VI:16 presented with an atrophy the cerebellar hemispheres (age, 63; duration of disease, 8 years).

On brain MRI, cerebellar atrophy was evident in all investigated family members with a cerebellar syndrome (Fig. 2). Conversely, patients without a cerebellar syndrome did not show cerebellar atrophy.

### 2.2 Results of the Molecular Analyses

A *DCTN1* mutation was detected in the previously reported brothers^12^ and in three of their children but was absent in the remaining affected and unaffected family members, including the two other patients with ALS/FTD, and thus was no longer considered as the cause of the disease. Furthermore, we did not detect linkage to or any mutation in the other tested ALS/FTD genes/loci despite the fact that all family members with ALS/FTD tested positive for a repeat expansion in the *C9ORF72* gene (Fig. 1). By Southern blot analysis, the repeat size was estimated to be 1300 to 11,200 repeats (Table 1). This also included a patient who had additional cerebellar ataxia. Thus, the cause of ALS/FTD in this family was elucidated as being attributed to *C9ORF72* repeat expansion but did not explain the ataxia phenotype in the other affected family members.

In the search for the genetic cause of the ataxia phenotype, we could not find any pathogenic repeat expansion in the 11 tested SCA genes. Likewise, point mutations in exon 4 of the *PRKCG* gene were excluded as well as linkage to the other four investigated gene loci. A genome‐wide linkage analysis with subsequent fine mapping revealed a shared chromosomal region on chromosome 1 between markers D1S2690 and D1S2737 (chr1:57,055,539‐61,426,261; hg19). Linkage analysis revealed a maximum two‐point LOD score of 3.3 at marker D1S2662 and a maximum multipoint LOD score of 4.4 at marker D1S2752 (Fig. S1). Genome sequencing did not suggest any candidate variant in the coding region of the genome. When focusing on the linked region on chromosome 1, only a single nucleotide variant was detected in a deep intergenic region and shared by all patients with SCA. This variant (chr1:59,573,414G > A, hg19) was located between the genes *LOC100131060* (distance 208 kb) and *HSD52* (distance 24 kb), thus not a convincing candidate variant for the SCA phenotype. We next focused on possible repeat expansions. Interestingly, a locus for a SCA phenotype (*SCA37*) had been mapped to a 2.8 Mb‐region on chromosome 1p32,^29^ a region within the linkage peak in our family. Notably, a heterozygous (ATTTC)_n_ insertion within an ATTTT repeat at position 57,832,716 on chromosome 1 (hg19) in the *DAB1* gene was demonstrated as the cause of ATX‐DAB1 at the *SCA37* locus.^9^ ExpansionHunter and exSTRa analyses for a *DAB1* repeat expansion in the genome data from our family demonstrated possible expansion of the ATTTT motif in the *DAB1* gene, but no evidence of the ATTTC insertion that had previously been reported in ATX‐DAB1. Manual filtering of the bam file for reads containing an ATTTC motif provided evidence of enrichment of this motif on a genome‐wide level. However, these reads could not be mapped to a specific genomic region because of the nature of short‐read sequencing (the ATTTC repeat in *DAB1* is deeply embedded in the expanded reference ATTTT motif).

**FIG 3:**
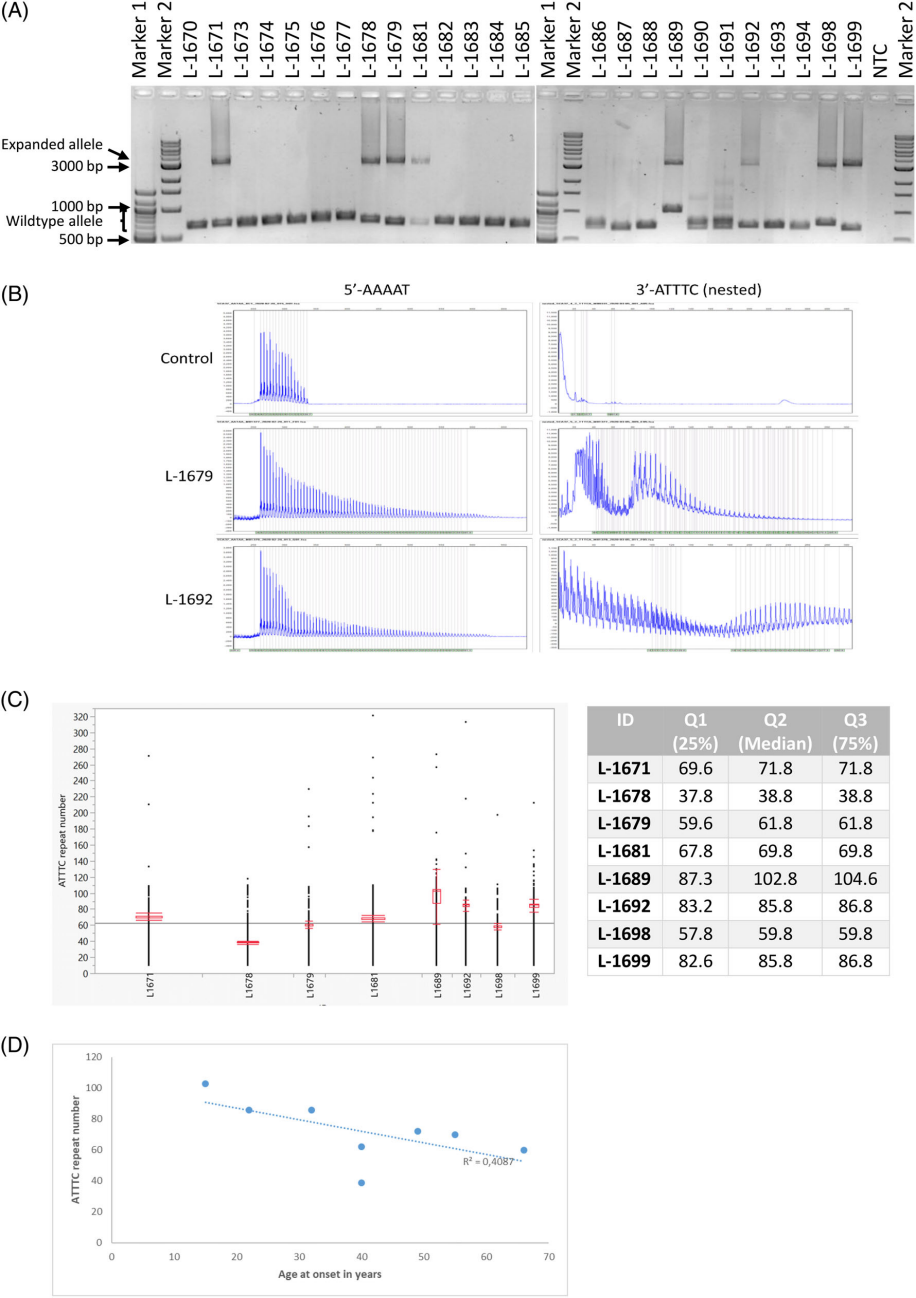
Repeat expansion/insertion in *DAB1*. (**A**) Results of the repeat‐spanning long‐range polymerase chain reaction (PCR) for all available family members. The PCR products were separated on agarose gel. The lower band (about 700 bp) indicates the nonexpanded wild‐type allele, which shows some degree of variability. The upper band (about 3500 bp) indicates the expanded repeat in affected individuals. The size seems to be comparable in all patients. (**B**) Results for the repeat‐primed PCR for the ATTTT motif (left panel) and the ATTTC motif (right panel) demonstrates expanded alleles in both patients (L numbers) when compared with a control. (**C**) Size determination of the ATTTC repeat. Analysis of the Nanopore run using all eight available samples and the EXP‐NBD112 chemistry is shown. The different calls for the ATTTC size are indicated by dots per individual, and the median (green line) and Quartile 1‐3 (Q1‐Q3, red box) are indicated. Red lines provide the range for 10% (lower line) and 90% (upper line) of the called repeat size. Numbers for Quartiles Q1, Q2, and Q3 are also provided in the table. (**D**) Moderate negative correlation of the ATTTC repeat number and age at onset of the spinocerebellar ataxia phenotype. The Pearson correlation coefficient *R* ^2^ is indicated. NTC, no template control. [Color figure can be viewed at wileyonlinelibrary.com]

We next performed a repeat‐spanning LR‐PCR to confirm the presence of an expanded sequence. We indeed demonstrated that all patients with SCA carried an expanded allele of about 3500 base pairs corresponding to a repeat number of approximately 600 (including about 500 base pairs of flanking sequence). Furthermore, all patients showed a smaller wild‐type allele of variable size in the range of approximately 700 to 800 base pairs corresponding to 40 to 60 repeats (Fig. 3A). We also used RP‐PCR to demonstrate the presence of the ATTTT and the ATTTC motifs in the expansion carriers V:25 and VI:14 (Fig. 3B). Finally, using Nanopore sequencing of a repeat‐spanning PCR product with a read depth of >4000 to 70,000x (Table S1) further resolved the structure of the repeat. All samples showed a comparable repeat length of almost 3000 nucleotides and an ATTTC interruption starting at around 75 repeats (Fig. S3). The repeat consists of ≈40 to 100 ATTTC repeats flanked by ≈75 and ≈415 ATTTT repeats upstream and downstream, respectively ([ATTTT]_≈75_[ATTTC]_≈40‐100_[ATTTT]_≈415_) (Figs 3C and [Link], [Link]). Of note, the ATTTC repeat size varied among family members, and there was a moderate negative correlation, which means there is a tendency for higher repeat length to go with earlier AAO (Pearson correlation coefficient: *r*(6)= −0.6393, *P* = 0.088; Figs 3D and S2). Notably, calculation of the ATTTC repeat size was almost identical across the different sequencing runs, which were based on independently generated LR‐PCR products, performed using different chemistry and at different time points.

## 3 Discussion

We report detailed clinical and genetic results from a seven‐generation German family with autosomal dominant inheritance of ALS/FTD and SCA. Although the cause of the FTD/ALS phenotype is a GGGGCC hexanucleotide repeat expansion in the *C9ORF72* gene, the SCA phenotype results from an ATTTC pentanucleotide repeat insertion into a long ATTTT repeat in the *DAB1* gene.

ALS was rapidly progressive with a mean survival of 2.5 years, and several patients with SCA in this family have been living with the disease for >40 years. The patients with SCA have a rather homogenous clinical phenotype of slowly progressive SCA, in some cases accompanied by vertical nystagmus, dystonia, oculomotor symptoms with diplopia, cognitive decline, and tremor. All cerebral MRIs of patients with SCA showed cerebellar atrophy (cerebellar hemispheres and sometimes the upper part of vermis).

Although the co‐occurrence of ALS/FTD and SCA in our family is explained by two different genetic causes, *SETX* mutations encoding senataxin can lead to either juvenile ALS or a form of ataxia, that is, ataxia oculomotor apraxia type 2.^30^, ^31^ Another example for the co‐occurrence of ALS and SCA due to a seemingly shared genetic cause was reported in one member of an SCA family linked to *CACNA1A* mutations.^32^

Autosomal dominant repeat expansions in *C9ORF72* are the most common cause of familial ALS in Northern Europe and are well known to cause familial FTD.^33^, ^34^ However, because it was only identified recently, we missed this diagnosis for many years and initially attributed the disease to a missense variant in *DCTN1* ^*12*^ based on the presence of the mutation in two affected siblings and the link of *DCTN1* to Perry syndrome, a late‐onset, atypical form of Parkinson's disease.^35^ From a clinical point of view, the repeat expansion in *C9ORF72* and its variable expressivity fully explains the presence of pure motor neuron disease and FTD in the family. Survival in *C9ORF72*‐positive patients with ALS/FTD is reported to be shorter than in sporadic ALS,^26^ which was also observed in the present study.

In all patients with SCA who were available for testing, we found an expanded ATTTC/ATTTT repeat in the *DAB1* gene, leading to a diagnosis of ATX‐DAB1. ATX‐DAB1 is characterized by an adult‐onset pure cerebellar syndrome distinctly presenting with dysarthria and often altered vertical eye movements. Other features may include mild dysmetria in the upper extremities, dysphagia, and oscillopsia.^9^, ^10^ Clinical progression is reported as slow.^10^ In our family, slowly progressive, predominant gait ataxia was the main feature, and dysphagia and tremor were late symptoms as reported previously. Notably, vertical nystagmus could not be confirmed as a “red flag” of ATX‐DAB1 because it was only present in half of our patients. Furthermore, none of our patients with SCA reported oscillopsia. Constant double vision attributed to vertical bulbar deviation was reported in five patients only.

Interestingly, postmortem neuropathology studies in two patients with ATX‐DAB1 revealed a severe loss of Purkinje cells.^1^ No significant neuropathological alterations were identified in other brain regions in agreement with a pure cerebellar syndrome^1^ and the restriction of MRI abnormalities to the cerebellum.

The ATTTC repeat insertion is always flanked on both sites by ATTTT repeat expansions, which challenges its detection by standard tools, including RP‐PCR and ExpansionHunter detection applied to genome data. The existence of the ATTTC insertion in our family was confirmed with Nanopore sequencing, and the number of ATTTC repeats was in the range of previous reports^9^, ^27^ and slightly above. Of note, the ATTTC repeat insertion in the 5′ noncoding regulatory region of *DAB1* has been described as being unstable.^1^, ^9^ To determine the exact size of repeat expansions, especially in those that contain different motifs, is still challenging. Even newly available methods such as third‐generation sequencing have a considerable error rate, and accuracy is <100% although chemistry and bioinformatics tools are being constantly improved. Analyses of repetitive sequences is particularly prone to sequencing errors.^36^ We used the NCRF software to calculate the size of the ATTTC insertion in available family members in our study. We observed variable sizes between ≈39 and 103 ATTTC repeats, with a trend for a negative correlation with AAO (Fig. 3D). We were able to evaluate two transmissions: the repeat number was stable in one transmission despite earlier AAO (V:27 > VI:14) and expanded in another (V:29 > VI:16) despite a later AAO (Fig. S2). Thus, factors modifying the AAO in *DAB1* remain elusive.

Furthermore, although a pure repeat expansion such as in *C9ORF72* can be explained by flipping events of the polymerase, the occurrence of an expansion/insertion as in *DAB1* is more complex. Haplotype studies in unaffected chromosomes suggested that the primary mutational mechanism, leading to the (ATTTC)_n_ insertion, were likely one or more T > C substitutions in an (ATTTT)_n_ pure allele of approximately 200 repeats. Then, the (ATTTC)_n_ expanded in size, generating a deleterious allele in *DAB1* that leads to SCA37.^37^

Without genetic testing, the diagnosis of the specific type/genetic cause of ALS/FTD and SCA in a single patient remains challenging because it is difficult to distinguish different monogenic and sporadic forms of ALS/FTD or SCA on clinical grounds, although some “red flags” exist. In the vast majority of patients, ALS occurs as a sporadic motor neuron disease with the involvement of the upper and lower motor neurons characterized by rapid decline and death after 2 to 5 years. ALS and FTD in the same family are often related to an autosomal dominant repeat expansion in the *C9ORF72* gene, which is the most frequent and highly penetrant genetic cause in Caucasian ALS cases (≈40% of familial ALS).^38^ Patients with *C9ORF72*‐related ALS/FTD are reported to present at a younger AAO^22^ and have a shorter survival when compared with other patients. On the other hand, different forms of SCAs share the common features of gait ataxia and dysarthria, often in combination with ocular disorders, seizures, extrapyramidal signs, and deterioration of cognitive function. The reported phenotypic spectrum of ATX‐DAB1 resembles ATX‐ATXN3 (*SCA3* locus) and ATX‐TBP (*SCA17* locus). However, we previously excluded repeat expansions in these genes.

Our study has two lines of limitations. On the clinical site, it was challenging to distinguish patients at advanced stages of SCA from patients with FTD/ALS when they presented with additional nonataxia signs such as motor neuron involvement or dystonia. From a genetic point of view, despite applying the most recent technologies (short‐ and long‐read next sequencing), we could not precisely determine the size of the repeat and the ATTTC insertion, although there was a narrow range of the median repeat size across experiments but some intra experimental variability (Figs 3C, S4A and S5). This can, at least partly, be overcome by deep sequencing, that is, high coverage of the target region, and potentially by avoiding a PCR amplification step. Further technical improvement and more sophisticated analysis tools are needed to overcome these limitations.

Taken together, the work toward establishing the genetic diagnoses in this family taught us three important lessons. First, affected individuals in one family do not have to have the same genetic cause, and it is possible that different genetic diseases segregate within the same family and selectively affect different family members possibly leading to a blended phenotype. A family such as ours with a combination of different neurological signs and symptoms may point to an autosomal recessive form of ataxia that often presents with a broad phenotypic spectrum as is the case in Friedreich's ataxia.^39^ However, our family clearly showed a dominant mode of inheritance with heterozygous pathogenic repeat expansions. To our knowledge, this is the first large family known to have two different disease‐causing repeat expansions. Second, candidate gene analyses require regular updates enabling testing of newly identified genetic causes for overlapping disorders. None of the genes mutated in our family appeared on the respective gene lists when we started to investigate this family about 2 decades ago. Third, even in the era of whole‐genome sequencing, comprehensive analysis is warranted, including repeat expansion screening in neurodegenerative disorders, despite the lack of clear anticipation. Of note, we report the first German family with ATX‐DAB1, and it is likely that there are other patients with ATX‐DAB1 who are currently awaiting a diagnosis. Based on the identification of the genetic cause in our family, we are now in the position to provide meaningful counseling to the offspring in the sixth and seventh generations.

## Author Roles

(1) Conception and Design of the Study, (2) Acquisition and Analysis of Data, (3) Drafting a Significant Portion of the Manuscript or Figures, (4) Reviewing and Editing the Manuscript.

A.R.: 1, 2, 3

H.P.: 2, 3

M.T.: 2, 3

H.R.: 2, 3

S.K.: 2, 4

S.Szymczak.: 2, 4

A.E.V.: 2, 4

K.M.: 2, 4

I.S.: 2, 4

J.H.W.: 2, 4

H.T.: 2, 4

P.S.: 2, 4

K.Z.: 2, 4

S.Schaake: 2, 4

A.W.: 2, 4

C.Z.: 2, 4

C.D.: 2, 4

J.T.: 2, 4

A.C.L.: 2, 4

C.K.: 1, 2

M.B.: 2, 3

K.L.: 1, 2, 3

## Financial Disclosures

A.R. and A.C.L. report employment with the University of Ulm. A.R. received a grant from the DGM e.V. (German Society for Muscle Patients) and reports consultancy for Ergomed, Sanofi and Argenx H.P. reports employment with the University Hospital Marburg. M.T. and S. Schaake report employment with the University Hospital Schleswig‐Holstein, Campus Lübeck. H.R. reports a grant from the National Health and Medical Research Council EL1 Fellowship Funding and employment with The University of Melbourne. S.K. and C.D. report employment with the University Hospital Essen. S. Szymczak and C.Z. report employment with the University of Lübeck. A.E.V. reports employment with the University Medical Center Hamburg‐Eppendorf. K.M. is a member of the Committee Neurogenetics of the German Network for Motor Neuron Diseases and reports employment with the University of Ulm. I.S. reports grants from the Fundação para a Ciência e a Tecnologia and Ministério da Ciência, Tecnologia e Ensino Superior (Portugal), Project PTDC/MED‐GEN/29255/2017, and employment with the Universidade do Porto. J.H.W. reports employment with University Medicine Mannheim. H.T. reports employment with the Christian‐Albrechts‐University Kiel. P.S. reports employment with the University Hospital Schleswig‐Holstein, Campus Lübeck. K.Z. reports a neurological practice in Munich. A.W. is a consultant for medical writing to CENTOGENE GmbH and reports a grant from the German Research Foundation (FOR2488) and employment with the University of Ulm. J.T. reports grants from the German Research Foundation (TR1714/4–1), Peter and Traudl Engelhorn Stiftung, and Canadian Institute of Health Research and employment with the University Hospital of Schleswig‐Holstein, Campus Lübeck. C.K. is a medical advisor to Centogene for genetic testing of movement disorders excluding Parkinson's disease and reports a consultancy with the Scientific Advisory Board of Retromer Therapeutics; honoraria from the Scientific Advisory Board of the Else Kroener Fresenius Foundation; grants from the German Research Foundation, the Bundesministerium fuer Bildung und Forschung (BMBF), The Michael J. Fox Foundation, the International Parkinson and Movement Disorder Society, and the European Community; intramural funds from the University of Lübeck; employment with the University of Lübeck; and royalties from Oxford University Press. M.B. reports advisory boards of the Australian Academy of Health and Medical Sciences Australian Learned Academies Data Internetworking Network Project Steering Committee (member), Clinical Genomics Advisory Committee, Kinghorn Sequencing Center (chair), Gen V Scientific Advisory Committee, Murdoch Children's Research Institute (member), Murdoch Children's Research Institute Big Data Review Panel (invited panel member), and Viertel Foundation Medical Advisory Board (member); honoraria from an examination of a thesis in Australia; NHMRC Investigator Grant APP1195236; and employment with the University of Melbourne. K.L. reports grants from the German Research Foundation, Movement Disorders Society, Damp Foundation, and The Michael J. Fox Foundation (GP2 project) and employment with the University of Lübeck.

## Supporting information


**Figure S1.** Results of linkage analysis in the linked region on chromosome 1. (**A**) The plot of the two‐point logarithm of the odds (LOD) score using FASTLINK over the 15 investigated short tandem repeat markers is shown, analyzing the family of II:3 as one family. (**B**) The plot of the multipoint LOD score using SimWalk is shown. The blue line indicates data analyzing the family of II:3 as one family, and the red line is based on separating the family in two branches (based on ancestors III:6 and III:8. (**C**) The LOD scores of both analyses for each marker are shown. Highest LOD scores are highlighted in bold.

**Figure S2.** ATTTC repeat number and age at onset of spinocerebellar ataxia (SCA). The pedigree of the family is shown with information on ATTTC repeat numbers (in red, based on the second Nanopore run) and age at onset (in green) of patients with SCA. Squares and circles represent males and females, respectively. Dashed symbols indicate individuals who are deceased. Clinical information is indicated as described in the legend.

**Figure S3.** Location of the ATTTC interruption within the ATTTT repeat. Results of Nanopore sequencing using the native barcoding kit EXP‐NBD112 are shown. The long‐range polymerase chain reaction product spanning the repeat in *DAB1* was sequenced and analyzed using Noise‐Cancelling Repeat Finder. The graphs illustrate the presence of the ATTTT repeat (blue line) and substitutions within this motif to ATTTC (high orange line). Results are shown for all eight available carriers. All patients showed a comparable repeat length of about 3000 nucleotides (from position ≈400–3350) and an ATTTC interruption starting at position ≈750 (≈350 base pairs in the repeat region, corresponding to repeat number ≈75). The width of the ATTTC interruptions (high orange peak) seems to be variable (≈200–500 base pairs, corresponding to ≈40–100 ATTTC repeats).

**Figure S4.** Size determination of the ATTTC repeat. (**A**) Analysis of the third Nanopore run using all eight available samples and the EXP‐NBD104 chemistry. The different calls for the ATTTC size are indicated by dots per individual, and the median (green line) and Quartile 1‐3 (Q1‐Q3, red box) are indicated. Numbers are also provided in the table. (**B**) High‐resolution agarose gel of the expanded allele after long‐range polymerase chain reaction reveals slight differences in the repeat size. (**C**) Screenshot of Integrative genome viewer (IGV) for sample L‐1671 using EXP‐NBD112 as an example for the high number of calls and the occasionally visible repeat expansions (unfiltered data).

**Figure S5.** Size determination of the ATTTT repeat. Analysis of the Nanopore run using all eight available samples and the EXP‐NBD104 chemistry. The different calls for the ATTTT size are indicated by dots per individual, and the median (green line) and Quarlite 1‐3 (Q1‐Q3, red box) are indicated. Numbers are also provided in the table. The repeat 5′ of the ATTTC repeat is shown in (**A**) and the 3′ region in (**B**).

**Table S1.** Nanopore statistics

**Appendix S1**: Supporting Information

**Segment 1**. Patient V:25 at the age of 68 years showing cerebellar ataxia of the legs more than the arms, a gaze‐evoked nystagmus, vertical nystagmus, and a deviation of gaze as described in the text. Dysarthria is of a mixed cerebellar and bulbar type. Flexion dystonia of the toes when walking is shown.

**Segment 2**. Patient VI:16 at the age of 55 years. Gaze‐evoked and fixation nystagmus without vertical nystagmus are shown.

## Data Availability

The data that support the findings of this study are available from the corresponding author upon reasonable request.
