# Supplementary material for: Familial Cerebellar Ataxia and Amyotrophic Lateral Sclerosis/Frontotemporal Dementia with DAB1 and C9ORF72 Repeat Expansions: An 18‐Year Study
Source: Mov Disord. 2022 Sep 23;37(12):2427–39. doi: 10.1002/mds.29221 (PMC10900262; doi:10.1002/mds.29221)
Supplement: Supplementary file 1 — Figure S1. Results of linkage analysis in the linked region on chromosome 1. (A) The plot of the two‐point logarithm of the odds (LOD) score using FASTLINK over the 15 investigated short tandem repeat markers is shown, analyzing the family of II:3 as one family. (B) The plot of the multipoint LOD score using SimWalk is shown. The blue line indicates data analyzing the family of II:3 as one family, and the red line is based on separating the family in two branches (based on ancestors III:6 and III:8. (C) The LOD scores of both analyses for each marker are shown. Highest LOD scores are highlighted in bold. [file MDS-37-2427-s004.pdf]

A **Two-point LOD score (FASTLINK)**

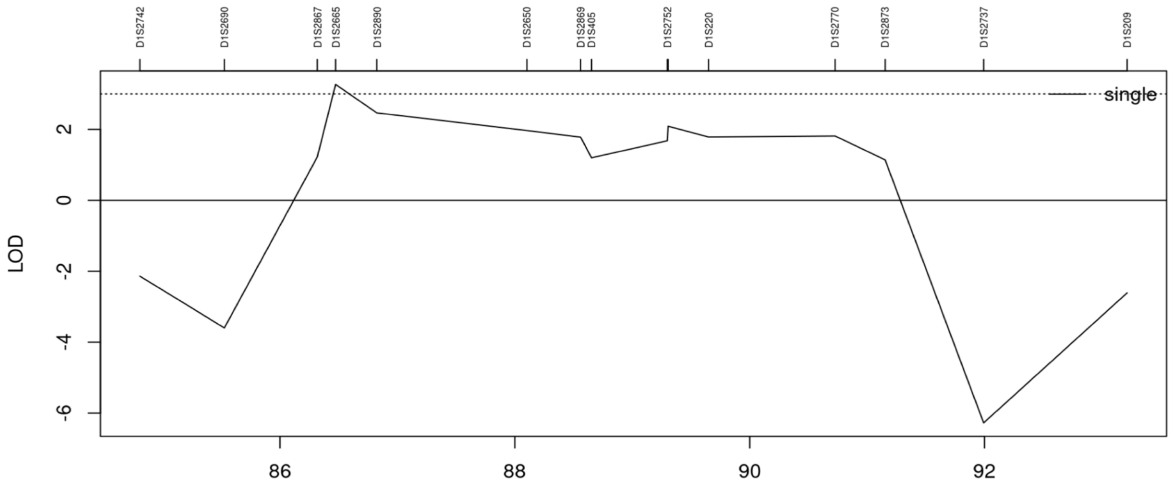

B **Multipoint LOD score (SIMWALK)**

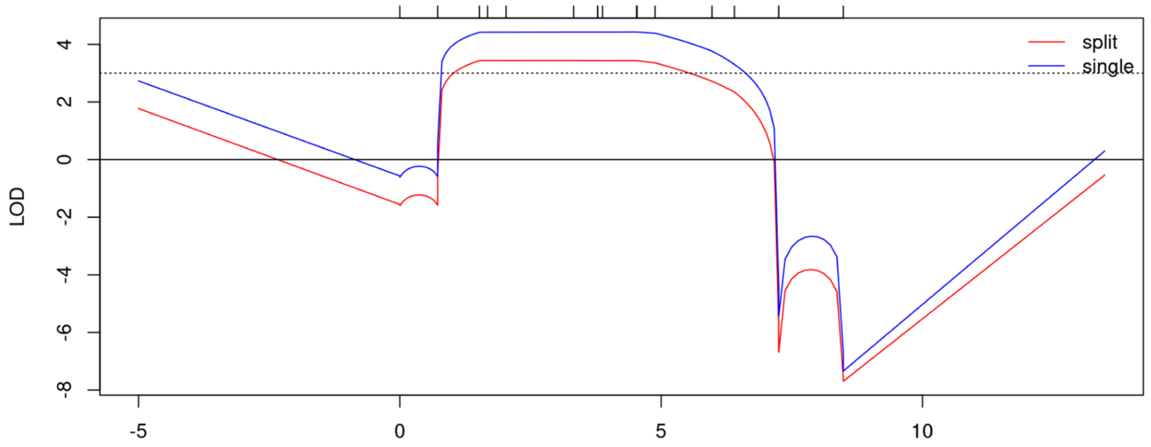

C **LOD scores of the two-point and multipoint analyses**

Marker	2-point FASTLINK	multipoint SIMWALK
D1S2742	-2.137	-0.590
D1S2690	-3.597	-0.020
D1S2867	1.221	4.418
D1S2665	**3.265**	4.422
D1S2890	2.462	4.422
D1S2650	1.967	4.426
D1S2869	1.782	4.425
D1S405	1.196	4.425
D1S2752	1.678	**4.429**
D1S2700	2.088	4.428
D1S220	1.786	4.384
D1S2770	1.814	3.763
D1S2873	1.137	3.301
D1S2737	-6.272	-4.942
D1S209	-2.609	-7.017

Figure S1: Results of linkage analysis in the linked region on chromosome 1. (A) The plot of the two-point LOD score using FASTLINK over the 15 investigated STR markers is shown analyzing the Family of II:3 as one family. (B) The plot of the multipoint LOD score using SIMWALK is shown. The blue line indicates data analyzing the Family of II:3 as one family, and the red line is based on separating the family into two branches (based on the ancestors III:6 and III:8). (C) The LOD scores of both analyses for each marker are shown. The highest LOD scores are highlighted in bold.
