# Supplementary material for: Familial Cerebellar Ataxia and Amyotrophic Lateral Sclerosis/Frontotemporal Dementia with DAB1 and C9ORF72 Repeat Expansions: An 18‐Year Study
Source: Mov Disord. 2022 Sep 23;37(12):2427–39. doi: 10.1002/mds.29221 (PMC10900262; doi:10.1002/mds.29221)
Supplement: Supplementary file 2 — Figure S2. ATTTC repeat number and age at onset of spinocerebellar ataxia (SCA). The pedigree of the family is shown with information on ATTTC repeat numbers (in red, based on the second Nanopore run) and age at onset (in green) of patients with SCA. Squares and circles represent males and females, respectively. Dashed symbols indicate individuals who are deceased. Clinical information is indicated as described in the legend. [file MDS-37-2427-s005.pdf]

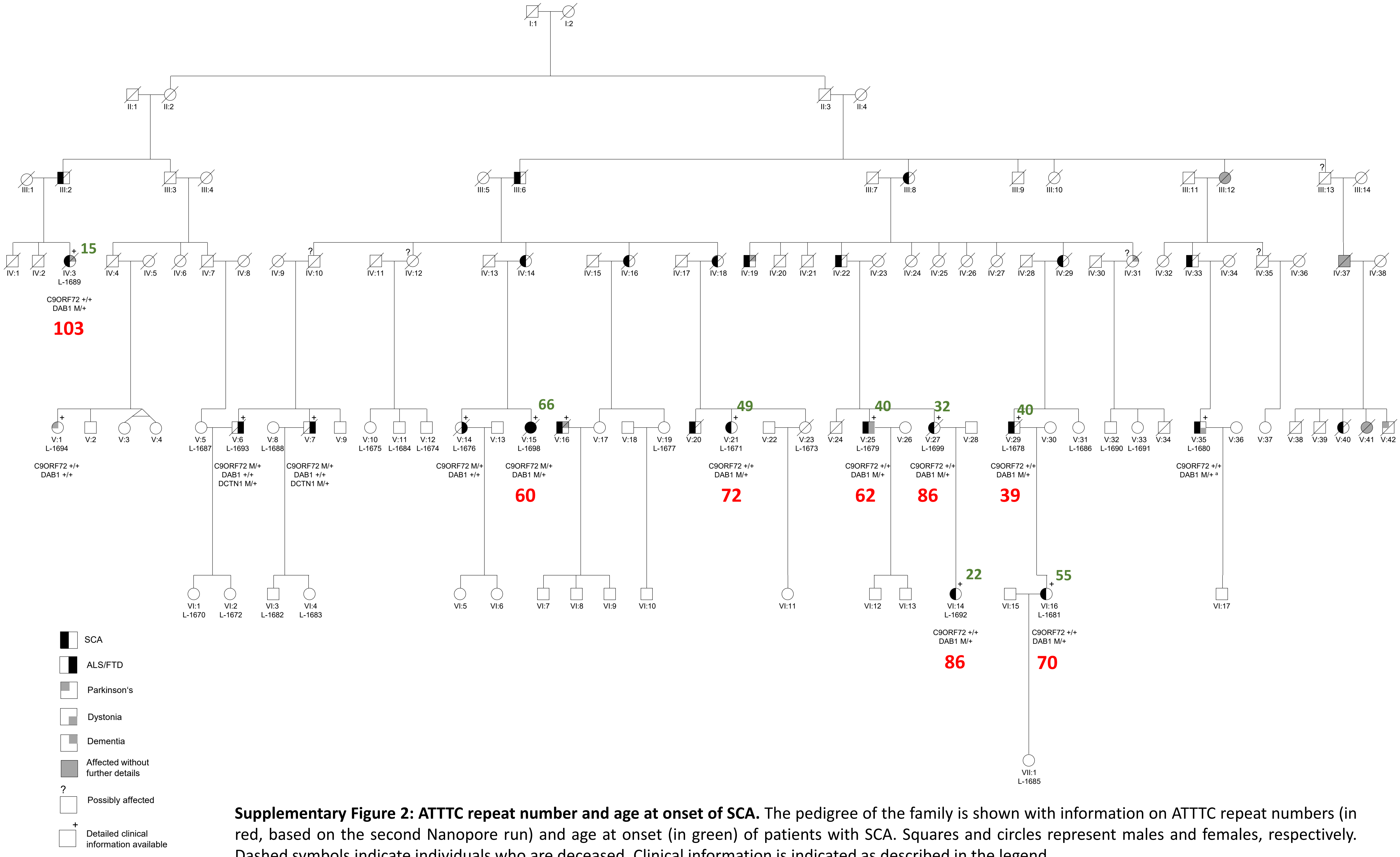

Supplementary Figure 2: ATTTC repeat number and age at onset of SCA. The pedigree of the family is shown with information on ATTTC repeat numbers (in red, based on the second Nanopore run) and age at onset (in green) of patients with SCA. Squares and circles represent males and females, respectively. Dashed symbols indicate individuals who are deceased. Clinical information is indicated as described in the legend.
