# Supplementary material for: Familial Cerebellar Ataxia and Amyotrophic Lateral Sclerosis/Frontotemporal Dementia with DAB1 and C9ORF72 Repeat Expansions: An 18‐Year Study
Source: Mov Disord. 2022 Sep 23;37(12):2427–39. doi: 10.1002/mds.29221 (PMC10900262; doi:10.1002/mds.29221)
Supplement: Supplementary file 3 — Figure S3. Location of the ATTTC interruption within the ATTTT repeat. Results of Nanopore sequencing using the native barcoding kit EXP‐NBD112 are shown. The long‐range polymerase chain reaction product spanning the repeat in DAB1 was sequenced and analyzed using Noise‐Cancelling Repeat Finder. The graphs illustrate the presence of the ATTTT repeat (blue line) and substitutions within this motif to ATTTC (high orange line). Results are shown for all eight available carriers. All patients showed a comparable repeat length of about 3000 nucleotides (from position ≈400–3350) and an ATTTC interruption starting at position ≈750 (≈350 base pairs in the repeat region, corresponding to repeat number ≈75). The width of the ATTTC interruptions (high orange peak) seems to be variable (≈200–500 base pairs, corresponding to ≈40–100 ATTTC repeats). [file MDS-37-2427-s006.pdf]

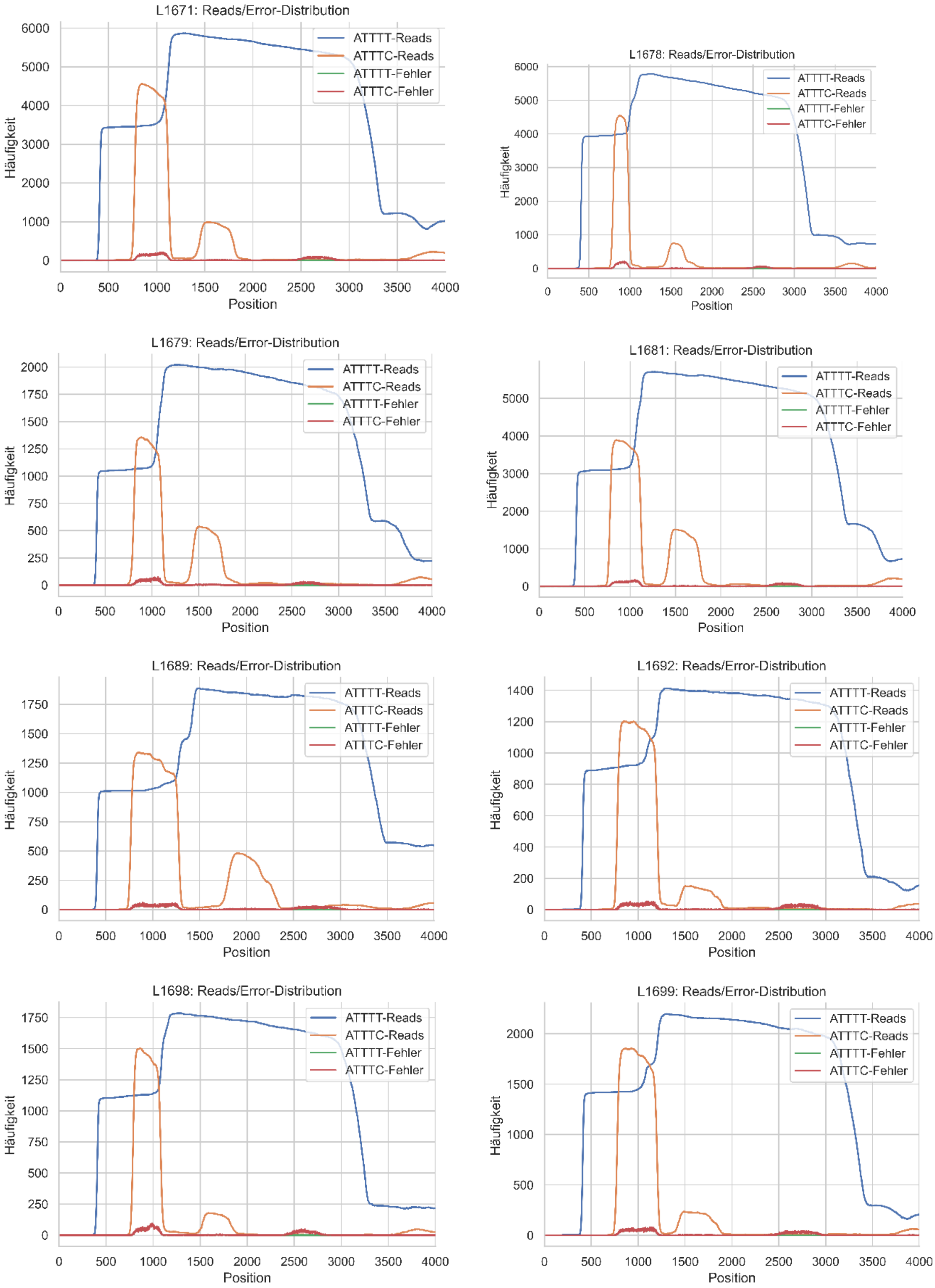

Supplementary Figure 3. Location of the ATTTC interruption within the ATTTT repeat. Results of Nanopore sequencing using the native barcoding kit EXP-NBD112-24 are shown. The long-range PCR product spanning the repeat in *DAB1* was sequenced and analyzed using NCRF. The graphs illustrate the presence of the ATTTT repeat (blue line) and substitutions within this motif to ATTTC (orange line). Results are shown for all eight available carriers. All patients showed a comparable repeat length of about 3000 nucleotides (from position ~400-3350) and an ATTTC interruption starting at position ~750 (~350 base pairs in the repeat region, corresponding to repeat number ~75-80. The width of the ATTTC interruptions (high orange peak) seems to be variable (~200-500 base pairs, corresponding to ~40-100 ATTTC repeats).
