# Supplementary material for: Familial Cerebellar Ataxia and Amyotrophic Lateral Sclerosis/Frontotemporal Dementia with DAB1 and C9ORF72 Repeat Expansions: An 18‐Year Study
Source: Mov Disord. 2022 Sep 23;37(12):2427–39. doi: 10.1002/mds.29221 (PMC10900262; doi:10.1002/mds.29221)
Supplement: Supplementary file 4 — Figure S4. Size determination of the ATTTC repeat. (A) Analysis of the third Nanopore run using all eight available samples and the EXP‐NBD104 chemistry. The different calls for the ATTTC size are indicated by dots per individual, and the median (green line) and Quartile 1‐3 (Q1‐Q3, red box) are indicated. Numbers are also provided in the table. (B) High‐resolution agarose gel of the expanded allele after long‐range polymerase chain reaction reveals slight differences in the repeat size. (C) Screenshot of Integrative genome viewer (IGV) for sample L‐1671 using EXP‐NBD112 as an example for the high number of calls and the occasionally visible repeat expansions (unfiltered data). [file MDS-37-2427-s008.pdf]

A Size of the ATTTC repeat based on the third Nanopore run

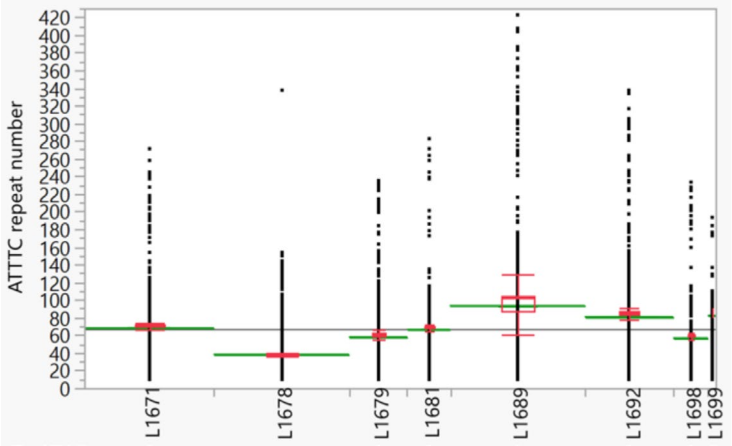

ID	Q1 (25%)	Q2 (Median)	Q3 (75%)
**L-1671**	69.8	70.8	71.8
**L-1678**	37.8	38.8	38.8
**L-1679**	58.8	60.8	61.8
**L-1681**	67.8	69.6	69.8
**L-1689**	86.8	101.8	103.8
**L-1692**	83.4	85.8	86.8
**L-1698**	57.8	59.4	59.8
**L-1699**	83.8	85.8	86.8

B Size of the repeat region based on long-range PCR

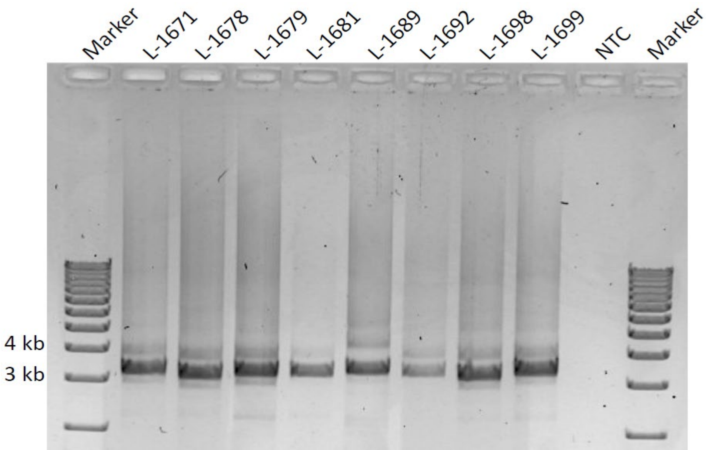

C IGV screenshot of repeat region

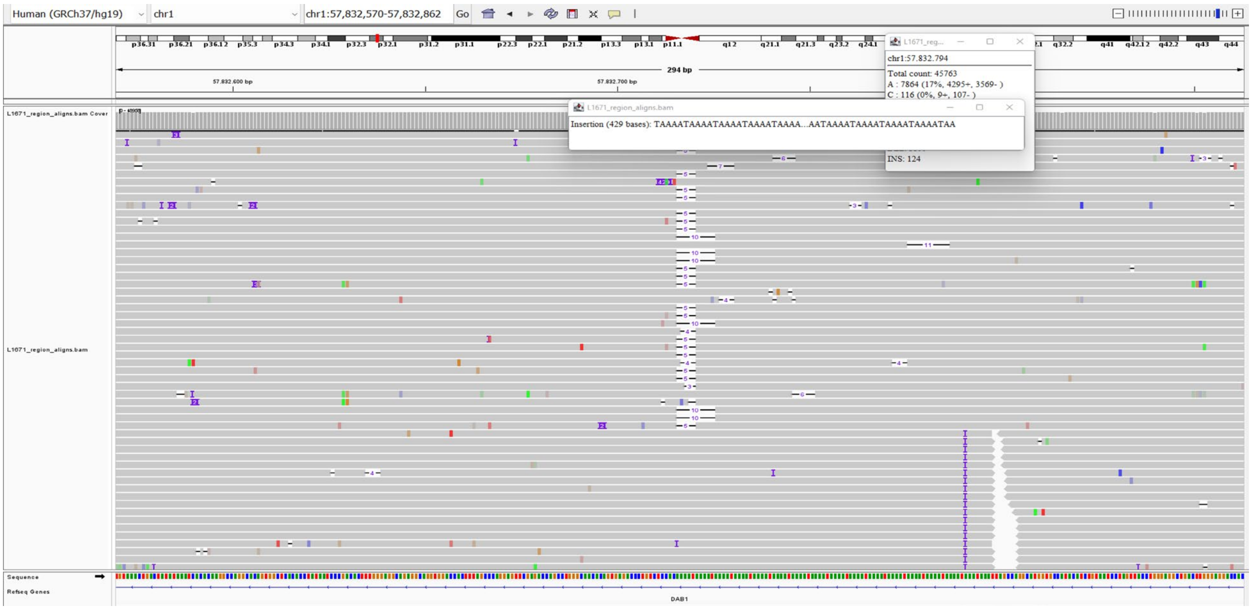

Figure S4: Size determination of the ATTTC repeat. (A) Analysis of the third Nanopore run using all 8 available samples and the EXP-NBD104 chemistry. The different calls for the ATTTC size are indicated by dots per individual, median (green line) and Q1-Q3 (red box) are indicated. Numbers are also provided in the table**. (B)** High resolution agarose gel of the expanded allele after long-range PCR reveals slight differences in the repeat size. **(C)** Screenshot of IGV for sample L-1671 using EXP-NBD112, as an example for the high number of calls and the occasionally visible repeat expansions (unfiltered data).
