# Supplementary material for: Familial Cerebellar Ataxia and Amyotrophic Lateral Sclerosis/Frontotemporal Dementia with DAB1 and C9ORF72 Repeat Expansions: An 18‐Year Study
Source: Mov Disord. 2022 Sep 23;37(12):2427–39. doi: 10.1002/mds.29221 (PMC10900262; doi:10.1002/mds.29221)
Supplement: Supplementary file 5 — Figure S5. Size determination of the ATTTT repeat. Analysis of the Nanopore run using all eight available samples and the EXP‐NBD104 chemistry. The different calls for the ATTTT size are indicated by dots per individual, and the median (green line) and Quarlite 1‐3 (Q1‐Q3, red box) are indicated. Numbers are also provided in the table. The repeat 5′ of the ATTTC repeat is shown in (A) and the 3′ region in (B). [file MDS-37-2427-s003.pdf]

A Size of the ATTTT repeat upstream of the ATTTC insertion

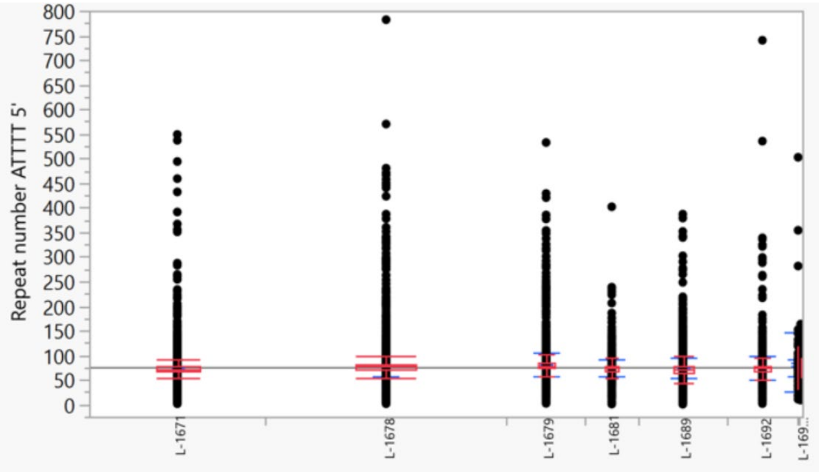

ID	Q1 (25%)	Q2 (Median)	Q3 (75%)
**L-1671**	67.8	72.8	78.0
**L-1678**	71.8	77.6	83.2
**L-1679**	74.0	79.4	85.2
**L-1681**	69.2	74.0	79.6
**L-1689**	65.4	72.2	79.4
**L-1692**	68.2	74.0	79.4
**L-1698**	64.6	76.6	87.6
**L-1699**	68.8	73.9	79.8

B Size of the ATTTT repeat downstream of the ATTTC insertion

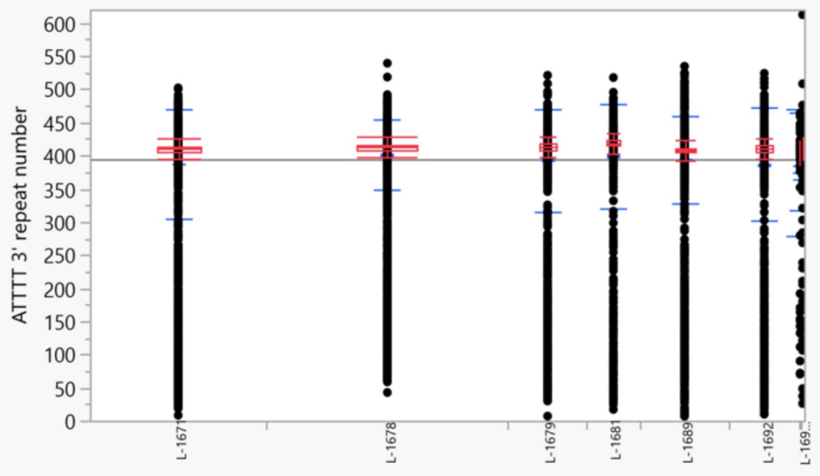

ID	Q1 (25%)	Q2 (Median)	Q3 (75%)
**L-1671**	406.2	410.2	413.8
**L-1678**	408.2	412.2	416.0
**L-1679**	409.2	413.4	417.4
**L-1681**	415.6	419.6	423.2
**L-1689**	404.4	408.2	412.0
**L-1692**	406.8	411.2	414.8
**L-1698**	402.2	409.0	414.5
**L-1699**	405.4	410.7	415.6

Figure S5: Size determination of the ATTTT repeat. Analysis of the Nanopore run using all 8 available samples and the EXP-NBD104 chemistry. The different calls for the ATTTT size are indicated by dots per individual, median (green line) and Q1-Q3 (red box) are indicated. Numbers are also provided in the table. The repeat 5´ of the ATTTC repeat is shown in **A** and the 3´ region in **B**.
