# Supplementary material for: Familial Cerebellar Ataxia and Amyotrophic Lateral Sclerosis/Frontotemporal Dementia with DAB1 and C9ORF72 Repeat Expansions: An 18‐Year Study
Source: Mov Disord. 2022 Sep 23;37(12):2427–39. doi: 10.1002/mds.29221 (PMC10900262; doi:10.1002/mds.29221)
Supplement: Supplementary file 7 — Appendix S1: Supporting Information [file MDS-37-2427-s009.docx]

*Case reports of examined patients with SCA*

Case IV:3 (SCA): This 83-year-old woman showed the following neurological signs after a disease course of 68 years: irregular horizontal smooth eye pursuit, slow horizontal dysmetric sakkades, no nystagmus, ataxia of gait, stance, limbs, cerebellar dysarthria and a 3-Hz tremor of the upper limbs and head. She had a five-year history of a dementing process. Reading or writing were no longer possible. Syntactic and phonological abilities were relatively preserved.

Case V:15 (ALS+SCA): This woman developed a cerebellar dysarthria at the age of 66 years, followed by gaze-evoked nystagmus and left-sided cerebellar ataxia with cerebellar atrophy. This was followed by a bulbar syndrome with an atrophic tetraparesis without upper motor neuron involvement less than one year later. A diagnosis of probable ALS was based on tongue fibrillations and acute and chronic denervation signs on EMG of both legs and the left arm without upper motor neuron signs. She died at the age of 69 years.

Case V:16 (SCA): This man presented with a slowly progressive cerebellar ataxia of the limbs, trunk and gait beginning at the age of 35 years with gaze-evoked and fixation nystagmus and cerebellar dysarthria. Diplopia occurred as part of an incomplete oculomotor palsy. Later, the patient developed a spastic tetraparesis and a dementing process with perseverations, euphoria, and disturbance of all mnestic functions, disorientation, mutism and psychosis. He died at the age of 67 years.

Case V:21 (SCA): This 62-year old woman developed a slowly progressive ataxia at the age of 49. At the age of 56, she showed cerebellar dysarthria, rebound, limb ataxia, but no nystagmus.

Case V:25 (SCA): This man developed ataxia at the age of 40 years. On neurological examination at the age of 68 years, we found cerebellar signs, gaze-evoked nystagmus and diplopia with mediocaudal deviation of the left eye without restriction in ocular motility. The dysarthria was of a combined bulbar and cerebellar type. In neuropsychological testing, deficits in visual short-term memory, episodic memory and attention deficits were obvious. Immediate and delayed recall were also impaired. Dystonic toe flexion was evident when walking.

Case V:27 (SCA): This woman developed a slowly progressive ataxia when she was pregnant at the age of 32 years. Until the age of 68, she presented with cerebellar ataxia of the limbs and dysarthria and was able to walk and do her activities of daily living unassisted. At the age of 69, she presented with upbeat nystagmus, gaze-evoked nystagmus, no double vision, cerebellar dysarthria and a generalized cerebellar syndrome. She died at age 69 after a 3 month history of left cerebral glioblastoma.

Case VI:14 (SCA): This 35-year old woman was diagnosed with nystagmus and ataxia from the age of 22 years. At the age of 35 years, she showed a cerebellar ataxia of the lower limbs without dysarthria, a gaze-evoked nystagmus (horizontal and vertical), and diplopia with mediocaudal deviation of the left eye without restriction in ocular motility.

Case V:29 (SCA): This 81-year old man reported slowly progressive ataxia beginning at the age of 40 years. On examination at the age of 81 years, he presented with cerebellar ataxia of the limbs, stance, gait and trunk, a proximal accentuated paresis of the arms>legs with dysphagia and atrophy of bulbar muscles, shoulder girdle and absent reflexes. He presented numbness in hand and feet and reduced vibration sense in the distal arms and legs equally. Additionally, there was diplopia with mediocaudal deviation of the left eye without restriction in ocular motility and loss of vibration sense. He died at the age of 82 years after having been bed-ridden for more than 10 years.

Case V:35 (SCA+dystonia)

In this male patient, first clinical signs appeared at the age of 27 years with writer’s cramp of one, and later, both arms. The dystonia spread to involve the legs. At the age of 68 years, he also showed cerebellar limb ataxia, generalized dystonia, and a combination of cerebellar and dystonic dysarthria and diplopia with mediocaudal deviation of the left eye with full range of eye movements.

Case IV:16 (SCA): At the age of 55 years, this woman developed a slowly progressive cerebellar ataxia of the limbs, trunk, gait and stance with gaze-evoked and fixation nystagmus. No vertical gaze paresis and no cerebellar dysarthria was apparent. Peripheral neurography was normal in the legs, but still pallesthesia was reduced, pointing to a posterior column involvement.
